# 3-(5-Methyl-3-phenyl-1*H*-pyrazol-1-yl)propanamide monohydrate

**DOI:** 10.1107/S160053681005035X

**Published:** 2010-12-08

**Authors:** Shu-Jiao Chen, Jian-Feng Zhang

**Affiliations:** a State Key Lab. Base of Novel Functional Materials and Preparation Science, Faculty of Materials Science and Chemical Engineering, Ningbo University, Ningbo, Zhejiang 315211, People’s Republic of China

## Abstract

In the title compound, C_13_H_15_N_3_O·H_2_O, the dihedral angle between the pyrazole and benzene rings is 26.6 (2)° and the N—C—C—C torsion angle is 153.6 (3)°. In the crystal, adjacent mol­ecules are linked by N—H⋯N, N—H⋯O and O—H⋯O hydrogen bonds into a network structure running along the *a* axis.

## Related literature

For the potential applications of substituted pyrazole derivatives as ligands, see: Shaw *et al.* (2004 ▶;) Pal *et al.* (2005 ▶). For the design and synthesis of various pyrazole ligands with special structural properties to fulfill the stereochemical requirements of the metal-binding sites, see: Bell *et al.* (2003 ▶); Paul *et al.* (2004 ▶) For pyrazole ligands with propanamide side-chains, see: Huang *et al.* (2009 ▶); Zhang *et al.* (2009 ▶).
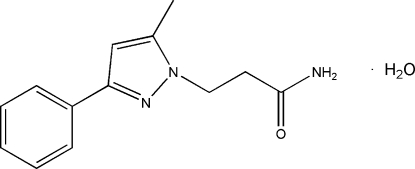


## Experimental

### 

#### Crystal data

C_13_H_15_N_3_O·H_2_O
*M* *_r_* = 247.30
Orthorhombic, 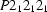

*a* = 6.5482 (13) Å
*b* = 12.609 (3) Å
*c* = 16.606 (3) Å
*V* = 1371.1 (5) Å^3^
*Z* = 4
Mo *K*α radiation
μ = 0.08 mm^−1^
*T* = 296 K
0.45 × 0.23 × 0.12 mm

#### Data collection

Rigaku R-AXIS RAPID diffractometer
Absorption correction: multi-scan (*ABSCOR*; Higashi, 1995 ▶) *T*_min_ = 0.977, *T*_max_ = 0.990
13414 measured reflections
1815 independent reflections
1323 reflections with *I* > 2σ(*I*)
*R*_int_ = 0.056

#### Refinement

*R*[*F* ^2^ > 2σ(*F* ^2^)] = 0.050
*wR*(*F* ^2^) = 0.140
*S* = 1.08
1815 reflections
164 parameters
H-atom parameters constrained
Δρ_max_ = 0.23 e Å^−3^
Δρ_min_ = −0.16 e Å^−3^

### 

Data collection: *RAPID-AUTO* (Rigaku, 1998) ▶; cell refinement: *RAPID-AUTO*; data reduction: *CrystalStructure* (Rigaku/MSC, 2004 ▶); program(s) used to solve structure: *SHELXS97* (Sheldrick, 2008 ▶); program(s) used to refine structure: *SHELXL97* (Sheldrick, 2008 ▶); molecular graphics: *ORTEP-3* (Farrugia, 1997 ▶); software used to prepare material for publication: *CrystalStructure*.

## Supplementary Material

Crystal structure: contains datablocks global, I. DOI: 10.1107/S160053681005035X/im2245sup1.cif

Structure factors: contains datablocks I. DOI: 10.1107/S160053681005035X/im2245Isup2.hkl

Additional supplementary materials:  crystallographic information; 3D view; checkCIF report

## Figures and Tables

**Table 1:** Hydrogen-bond geometry (Å, °)

*D*—H⋯*A*	*D*—H	H⋯*A*	*D*⋯*A*	*D*—H⋯*A*
N1—H1*A*⋯O2	0.86	2.03	2.867 (3)	165
N1—H1*B*⋯N3^i^	0.86	2.22	3.036 (3)	159
O2—H2*D*⋯O1^ii^	0.84	1.96	2.783 (3)	166.2
O2—H2*C*⋯O1^i^	0.85	2.03	2.872 (3)	168.5

Symmetry codes: (i) 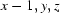; (ii) 
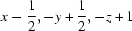
.

**Fig. 1.**
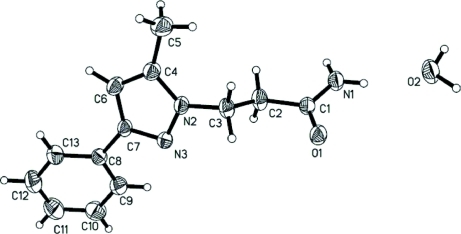
Molecular structure of the title compound. Displacement ellipsoids are drawn at the 30% probability level.

**Fig. 2.**
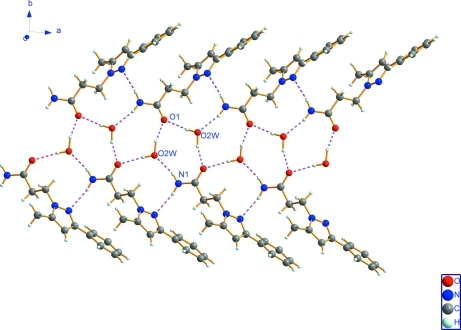
Interconnections between the organic and water molecules of the title compound. Dashed lines indicate N—H···O, N—H···N and O—H···O hydrogen bonds.

## supplementary crystallographic information

### Comment

In recent years, there has been considerable interest in the use of hemilabile ligands containing substituted pyrazole groups because of their potential applications in catalysis and their ability for complexes construction (Shaw *et al.*, 2004; Pal *et al.*, 2005). Nowadays, much attention has been focused on the design of various pyrazole ligands with special structural properties to fulfill the specific stereochemical requirement of a particular metal-binding site (Bell *et al.*, 2003; Paul *et al.*, 2004). Some new pyrazole ligands combined with propanamide side-chains were reported (Zhang *et al.*, 2009; Huang *et al.*, 2009). Here, we report another new *N*-pyrazolylpropanamide ligand, C_13_H_15_N_3_O . H_2_O, (Scheme 1).

As is shown in Figure 1, in the title compound, the dihedral angle between pyrazole ring and benzene ring is 26.6 (2)° and the torsion angle N3—C7—C8—C13 is 153.6 (3)°. In the crystal structure, there is a N—H···N hydrogen bond between two organic molecules. Additional O—H···O and N—H···O hydrogen-bonding interactions between the organic molecules and water produce a network structure (Figure 2). The hydrogen bonds in the network structure relate three organic molecules and one water molecule, where two O atoms in the O—H···O hydrogen bonds originate from two organic molecules while one N atom in the N—H···O hydrogen bond from a third organic molecule. The hydrogen bond geometry parameters are listed in Table 1.

### Experimental

A mixture of 5-methyl-3-phenyl-1*H*-pyrazole (1.58 g, 10 mmol), sodium hydroxide solution (2 mol/l, 2 ml) and *N*,*N*'-dimethylformamide (DMF) (50 ml) was stirred and heated to 333 K. A solution of acrylamide (1.0 g, 14 mmol) in DMF (10 ml) was added dropwise over a period of 15 minutes. The reaction was conducted by heating for 7 h at 333 K. The mixture was cooled to room temperature and filtered. Afterwards DMF was removed by vacuum distillation to give 1.94 g analytically pure 3-(5-methyl-3-phenyl-1*H*-pyrazol-1- yl)propanamide (yield: 85%; mp: 386 K). Recrystallization an acetonitrile water mixture in a 1:1 ratio yielded colorless single-crystals suitable for X-ray diffraction analysis. Analysis calculated for C_13_H_17_N_3_O_2_: C 63.14, H 6.93, N 16.99%; found: C 63.25, H 6.87, N 16.92%.

### Refinement

In the absence of significant anomalous dispersion effects, Friedel pairs were averaged. Atoms H2C and H2D (for H_2_O) were located in difference Fourier map and refined isotropically, with restrains of O2—H2C = 0.8506 Å, O2—H2D = 0.8424Å and H2C—O2—H2D = 104.5°. The remaining H atoms were positioned geometrically with N—H = 0.86Å (for NH_2_) and C—H = 0.93 (aromatic) or 0.96 (methyl) or 0.97Å (methylene) and constrained to ride on their parent atoms, with *U*_iso_(H) = *xU*_eq_(C,*N*), where *x* = 1.5 for methyl H and *x* = 1.2 for all other H atoms.

### Crystal data

**Table:**

C_13_H_15_N_3_O·H_2_O	*F*(000) = 528
*M**_r_* = 247.30	*D*_x_ = 1.198 Mg m^−^^3^
Orthorhombic, *P*2_1_2_1_2_1_	Mo *K*α radiation, λ = 0.71073 Å
Hall symbol: P 2ac 2ab	Cell parameters from 13460 reflections
*a* = 6.5482 (13) Å	θ = 3.2–27.4°
*b* = 12.609 (3) Å	µ = 0.08 mm^−^^1^
*c* = 16.606 (3) Å	*T* = 296 K
*V* = 1371.1 (5) Å^3^	Chip, colorless
*Z* = 4	0.45 × 0.23 × 0.12 mm

### Data collection

**Table:**

Rigaku R-AXIS RAPID diffractometer	1815 independent reflections
Radiation source: fine-focus sealed tube	1323 reflections with *I* > 2σ(*I*)
graphite	*R*_int_ = 0.056
ω scans	θ_max_ = 27.4°, θ_min_ = 3.2°
Absorption correction: multi-scan (*ABSCOR*; Higashi, 1995)	*h* = −8→7
*T*_min_ = 0.977, *T*_max_ = 0.990	*k* = −16→16
13414 measured reflections	*l* = −21→21

### Refinement

**Table:**

Refinement on *F*^2^	Secondary atom site location: difference Fourier map
Least-squares matrix: full	Hydrogen site location: inferred from neighbouring sites
*R*[*F*^2^ > 2σ(*F*^2^)] = 0.050	H-atom parameters constrained
*wR*(*F*^2^) = 0.140	*w* = 1/[σ^2^(*F*_o_^2^) + (0.0693*P*)^2^ + 0.2276*P*] where *P* = (*F*_o_^2^ + 2*F*_c_^2^)/3
*S* = 1.08	(Δ/σ)_max_ = 0.001
1815 reflections	Δρ_max_ = 0.23 e Å^−^^3^
164 parameters	Δρ_min_ = −0.16 e Å^−^^3^
0 restraints	Extinction correction: *SHELXL97* (Sheldrick, 2008), Fc^*^=kFc[1+0.001xFc^2^λ^3^/sin(2θ)]^-1/4^
Primary atom site location: structure-invariant direct methods	Extinction coefficient: 0.048 (6)

### Special details

**Table:**

Geometry. All e.s.d.'s (except the e.s.d. in the dihedral angle between two l.s. planes) are estimated using the full covariance matrix. The cell e.s.d.'s are taken into account individually in the estimation of e.s.d.'s in distances, angles and torsion angles; correlations between e.s.d.'s in cell parameters are only used when they are defined by crystal symmetry. An approximate (isotropic) treatment of cell e.s.d.'s is used for estimating e.s.d.'s involving l.s. planes.
Refinement. Refinement of *F*^2^ against ALL reflections. The weighted *R*-factor *wR* and goodness of fit *S* are based on *F*^2^, conventional *R*-factors *R* are based on *F*, with *F* set to zero for negative *F*^2^. The threshold expression of *F*^2^ > σ(*F*^2^) is used only for calculating *R*-factors(gt) *etc*. and is not relevant to the choice of reflections for refinement. *R*-factors based on *F*^2^ are statistically about twice as large as those based on *F*, and *R*- factors based on ALL data will be even larger.

### Fractional atomic coordinates and isotropic or equivalent isotropic displacement parameters (Å^2^)

**Table:**

	*x*	*y*	*z*	*U*_iso_*/*U*_eq_	
O1	0.1503 (3)	0.37278 (16)	0.44426 (14)	0.0598 (6)	
N3	0.6165 (4)	0.63188 (17)	0.33836 (14)	0.0455 (6)	
N2	0.4723 (4)	0.58241 (16)	0.29243 (14)	0.0456 (6)	
C7	0.7043 (4)	0.7022 (2)	0.28840 (17)	0.0432 (6)	
C1	0.0612 (4)	0.4538 (2)	0.42065 (18)	0.0451 (7)	
C3	0.3511 (4)	0.4985 (2)	0.32841 (19)	0.0505 (7)	
H3A	0.4382	0.4554	0.3625	0.061*	
H3B	0.2976	0.4534	0.2861	0.061*	
N1	−0.1361 (4)	0.4692 (2)	0.43135 (16)	0.0572 (7)	
H1A	−0.2084	0.4220	0.4556	0.069*	
H1B	−0.1926	0.5264	0.4141	0.069*	
C6	0.6151 (5)	0.6976 (2)	0.21225 (18)	0.0529 (7)	
H6A	0.6495	0.7386	0.1677	0.063*	
C8	0.8697 (5)	0.7707 (2)	0.31861 (18)	0.0463 (7)	
C2	0.1763 (5)	0.5407 (2)	0.3778 (2)	0.0578 (8)	
H2A	0.0830	0.5788	0.3428	0.069*	
H2B	0.2287	0.5905	0.4172	0.069*	
C4	0.4663 (5)	0.6208 (2)	0.21594 (18)	0.0509 (7)	
O2	−0.4388 (4)	0.32706 (19)	0.4938 (2)	0.0888 (10)	
H2D	−0.4329	0.2662	0.5146	0.168*	
H2C	−0.5655	0.3375	0.4857	0.092*	
C9	0.8839 (5)	0.7964 (3)	0.4002 (2)	0.0596 (8)	
H9A	0.7863	0.7708	0.4360	0.072*	
C12	1.1724 (6)	0.8742 (3)	0.2958 (3)	0.0849 (12)	
H12A	1.2706	0.9003	0.2605	0.102*	
C13	1.0158 (5)	0.8108 (2)	0.2666 (2)	0.0612 (9)	
H13A	1.0090	0.7952	0.2119	0.073*	
C10	1.0405 (6)	0.8592 (3)	0.4285 (2)	0.0767 (11)	
H10A	1.0489	0.8748	0.4832	0.092*	
C5	0.3188 (6)	0.5832 (3)	0.1551 (2)	0.0749 (11)	
H5A	0.2362	0.5278	0.1778	0.112*	
H5B	0.3913	0.5563	0.1092	0.112*	
H5C	0.2328	0.6410	0.1387	0.112*	
C11	1.1840 (7)	0.8988 (4)	0.3764 (3)	0.0923 (14)	
H11A	1.2884	0.9421	0.3955	0.111*	

### Atomic displacement parameters (Å^2^)

**Table:**

	*U*^11^	*U*^22^	*U*^33^	*U*^12^	*U*^13^	*U*^23^
O1	0.0465 (11)	0.0555 (11)	0.0775 (15)	0.0054 (11)	0.0067 (12)	0.0248 (11)
N3	0.0438 (12)	0.0434 (12)	0.0493 (13)	−0.0047 (11)	0.0011 (11)	0.0076 (10)
N2	0.0465 (13)	0.0423 (11)	0.0479 (13)	−0.0057 (11)	0.0041 (12)	0.0079 (10)
C7	0.0445 (14)	0.0357 (12)	0.0495 (15)	−0.0022 (12)	0.0054 (14)	0.0087 (12)
C1	0.0418 (15)	0.0457 (14)	0.0480 (16)	0.0001 (13)	0.0033 (13)	0.0050 (13)
C3	0.0473 (15)	0.0414 (13)	0.0628 (18)	−0.0064 (14)	0.0083 (15)	0.0044 (13)
N1	0.0415 (13)	0.0558 (14)	0.0742 (18)	0.0034 (12)	0.0085 (14)	0.0194 (13)
C6	0.0634 (18)	0.0479 (14)	0.0475 (16)	−0.0078 (15)	0.0016 (16)	0.0092 (13)
C8	0.0443 (14)	0.0376 (12)	0.0569 (17)	−0.0012 (12)	0.0000 (15)	0.0088 (12)
C2	0.0488 (17)	0.0455 (14)	0.079 (2)	0.0016 (15)	0.0151 (17)	0.0110 (15)
C4	0.0569 (17)	0.0487 (14)	0.0470 (15)	−0.0042 (15)	0.0000 (15)	0.0032 (13)
O2	0.0472 (13)	0.0764 (16)	0.143 (3)	−0.0005 (12)	0.0038 (16)	0.0493 (17)
C9	0.0590 (19)	0.0586 (17)	0.0611 (19)	−0.0053 (17)	0.0007 (17)	0.0017 (15)
C12	0.060 (2)	0.102 (3)	0.092 (3)	−0.030 (2)	0.008 (2)	0.011 (2)
C13	0.0547 (18)	0.0627 (18)	0.066 (2)	−0.0080 (17)	0.0053 (17)	0.0067 (16)
C10	0.073 (2)	0.084 (2)	0.074 (2)	−0.014 (2)	−0.014 (2)	−0.007 (2)
C5	0.086 (3)	0.078 (2)	0.060 (2)	−0.019 (2)	−0.014 (2)	0.0029 (18)
C11	0.069 (3)	0.109 (3)	0.098 (3)	−0.033 (3)	−0.011 (2)	−0.003 (3)

### Geometric parameters (Å, °)

**Table:**

O1—C1	1.240 (3)	C2—H2A	0.9700
N3—C7	1.344 (3)	C2—H2B	0.9700
N3—N2	1.365 (3)	C4—C5	1.476 (4)
N2—C4	1.360 (4)	O2—H2D	0.8424
N2—C3	1.451 (3)	O2—H2C	0.8505
C7—C6	1.394 (4)	C9—C10	1.378 (5)
C7—C8	1.473 (4)	C9—H9A	0.9300
C1—N1	1.319 (4)	C12—C11	1.376 (6)
C1—C2	1.509 (4)	C12—C13	1.388 (5)
C3—C2	1.505 (4)	C12—H12A	0.9300
C3—H3A	0.9700	C13—H13A	0.9300
C3—H3B	0.9700	C10—C11	1.371 (6)
N1—H1A	0.8600	C10—H10A	0.9300
N1—H1B	0.8600	C5—H5A	0.9600
C6—C4	1.375 (4)	C5—H5B	0.9600
C6—H6A	0.9300	C5—H5C	0.9600
C8—C13	1.385 (4)	C11—H11A	0.9300
C8—C9	1.397 (4)		
			
C7—N3—N2	104.6 (2)	C3—C2—H2B	109.1
C4—N2—N3	112.3 (2)	C1—C2—H2B	109.1
C4—N2—C3	128.9 (3)	H2A—C2—H2B	107.9
N3—N2—C3	118.8 (2)	N2—C4—C6	105.8 (3)
N3—C7—C6	110.7 (2)	N2—C4—C5	122.9 (3)
N3—C7—C8	119.4 (2)	C6—C4—C5	131.3 (3)
C6—C7—C8	129.9 (2)	H2D—O2—H2C	104.5
O1—C1—N1	122.7 (3)	C10—C9—C8	120.9 (3)
O1—C1—C2	120.8 (3)	C10—C9—H9A	119.6
N1—C1—C2	116.5 (3)	C8—C9—H9A	119.6
N2—C3—C2	112.5 (2)	C11—C12—C13	120.7 (4)
N2—C3—H3A	109.1	C11—C12—H12A	119.6
C2—C3—H3A	109.1	C13—C12—H12A	119.6
N2—C3—H3B	109.1	C8—C13—C12	120.1 (3)
C2—C3—H3B	109.1	C8—C13—H13A	119.9
H3A—C3—H3B	107.8	C12—C13—H13A	119.9
C1—N1—H1A	120.0	C11—C10—C9	120.3 (4)
C1—N1—H1B	120.0	C11—C10—H10A	119.9
H1A—N1—H1B	120.0	C9—C10—H10A	119.9
C4—C6—C7	106.6 (3)	C4—C5—H5A	109.5
C4—C6—H6A	126.7	C4—C5—H5B	109.5
C7—C6—H6A	126.7	H5A—C5—H5B	109.5
C13—C8—C9	118.4 (3)	C4—C5—H5C	109.5
C13—C8—C7	120.6 (3)	H5A—C5—H5C	109.5
C9—C8—C7	121.0 (3)	H5B—C5—H5C	109.5
C3—C2—C1	112.4 (2)	C10—C11—C12	119.6 (4)
C3—C2—H2A	109.1	C10—C11—H11A	120.2
C1—C2—H2A	109.1	C12—C11—H11A	120.2

### Hydrogen-bond geometry (Å, °)

**Table:**

*D*—H···*A*	*D*—H	H···*A*	*D*···*A*	*D*—H···*A*
N1—H1A···O2	0.86	2.03	2.867 (3)	165.
N1—H1B···N3^i^	0.86	2.22	3.036 (3)	159.
O2—H2D···O1^ii^	0.84	1.96	2.783 (3)	166.2
O2—H2C···O1^i^	0.85	2.03	2.872 (3)	168.5

Symmetry codes: (i) *x*−1, *y*, *z*; (ii) *x*−1/2, −*y*+1/2, −*z*+1.
